# Anthropometry, dietary intake, physical activity and sitting time patterns in adolescents aged 15–17 years: an international comparison in eight Latin American countries

**DOI:** 10.1186/s12887-020-1920-x

**Published:** 2020-01-21

**Authors:** Gerson Luis de Moraes Ferrari, Irina Kovalskys, Mauro Fisberg, Georgina Gomez, Attilio Rigotti, Lilia Yadira Cortés Sanabria, Martha Cecilia Yépez García, Rossina Gabriella Pareja Torres, Marianella Herrera-Cuenca, Ioná Zalcman Zimberg, Viviana Guajardo, Michael Pratt, Agatha Nogueira Previdelli, Shaun Scholes, Carlos A. Celis-Morales, Dirceu Solé, on behalf of the ELANS Study Group, Mauro Fisberg, Georgina Gómez Salas, Attilio Rigotti, Lilia Yadira Cortés Sanabria, Martha Cecilia Yépez García, Rossina Gabriella Pareja Torres, Marianella Herrera-Cuenca, Berthold Koletzko, Luis A. Moreno, Michael Pratt, Ioná Zalcman Zimberg, Irina Kovalskys, Viviana Guajardo, María Paz Amigo, Ximena Janezic, Fernando Cardini, Myriam Echeverry, Martin Langsman, Natasha Aparecida Grande de França, Guadalupe Echeverría, Leslie Landaeta, Óscar Castillo, Luz Nayibe Vargas, Luisa Fernanda Tobar, Yuri Milena Castillo, Georgina Gómez Salas, Rafael Monge Rojas, Anne Chinnock, Mónica Villar Cáceres, María Belén Ocampo, Rossina Pareja Torres, María Reyna Liria, Krysty Meza, Mellisa Abad, Mary Penny, Maritza Landaeta, Betty Méndez, Maura Vasquez, Omaira Rivas, Carmen Meza, Servando Ruiz, Guillermo Ramirez, Pablo Hernández, Alexandre D. P. Chiavegatto Filho, Priscila Bezerra Gonçalves, Claudia Alberico, Gerson Luis de Moraes Ferrari

**Affiliations:** 1 Centro de Investigación en Fisiología del Ejercicio - CIFE, Universidad Mayor, Santiago, Chile; 2 Departamento de Pediatria da Universidade Federal de São Paulo, São Paulo, Brazil; 3 Commitee of Nutrition and Wellbeing, International Life Science Institute (ILSI-Argentina), Buenos Aires, Argentina; 4 Instituto Pensi, Fundação José Luiz Egydio Setubal, Hospital Infantil Sabará, São Paulo, Brazil; 5 Departamento de Bioquímica, Escuela de Medicina, Universidad de Costa Rica, San José, Costa Rica; 6 Centro de Nutrición Molecular y Enfermedades Crónicas, Departamento de Nutrición, Diabetes y Metabolismo, Escuela de Medicina, Pontificia Universidad Católica, Santiago, Chile; 7 Departamento de Nutrición y Bioquímica, Pontificia Universidad Javeriana, Bogotá, Colombia; 8 Colégio de Ciencias de la Salud, Universidad San Francisco de Quito, Quito, Ecuador; 9 Instituto de Investigación Nutricional, La Molina, Lima, Peru; 10 Centro de Estudios del Desarrollo, Universidad Central de Venezuela (CENDES-UCV)/Fundación Bengoa, Caracas, Venezuela; 11 Departamento de Psicobiologia, Universidade Federal de São Paulo, São Paulo, Brazil; 12 Institute for Public Health, University of California San Diego, La Jolla, CA USA; 13 Faculdade de Ciências Biológicas e da Saúde, Universidade São Judas Tadeu, São Paulo, Brazil; 14 Institute of Epidemiology and Health Care, University College London, London, UK; 15 Grupo de Estudio en Educación, Actividad Física y Salud (GEEAFyS), Universidad Católica del Maule, Talca, Chile

**Keywords:** Obesity, Anthropometry, Sedentary behaviours, Physical activity, Energy intake, Macronutrients, Total fat

## Abstract

**Background:**

Although there is high prevalence of obesity and other cardiovascular risk factors among Latin American adolescents, there is limited evidence on dietary intake and physical activity (PA) patterns in this population. Therefore, we characterized anthropometry, dietary intake, PA and sitting time (ST) in adolescents aged 15–17 years from eight Latin American countries.

**Methods:**

Six hundred seventy-one adolescents (41.4% girls) from the Latin American Study of Nutrition and Health (ELANS) were included. Nutritional status was classified by four BMI (kg/m^2^) categories. Waist circumference (WC) was categorized as above or below thresholds. Dietary intake was assessed through two non-consecutive 24-h dietary recalls. PA and ST were measured using the International Physical Activity Questionnaire (IPAQ). We calculated overall and country-specific estimates by sex and tested for differences between boys and girls.

**Results:**

Differences in the prevalence of overweightness (15.1 and 21.6%) and obesity (8.5 and 6.5%) between boys and girls, respectively, were statistically insignificant (*p* = 0.059). Average energy intake was 2289.7 kcal/day (95% CI: 2231–2350) for boys and 1904.2 kcal/day (95% CI: 1840–1963) for girls (*p* < 0.001). In relation to macronutrient intake for boys and girls, respectively, the average intake (expressed as percentage of total energy) was 15.0 and 14.9% for protein; 55.4 and 54.9% for carbohydrates; 14.1 and 14.5% for added sugar; 29.5 and 30.1% for total fat; and 9.6 and 9.9% for saturated fat (*p* > 0.05 for all outcomes). There was no statistically significant difference in the prevalence of total energy (TE) saturated fat and added sugar (>10% of TE) between girls and boys (49.6% versus 44.8 and 81.7% versus 76.1%, respectively). Prevalence of physical inactivity was 19% in boys and 43.7% in girls (*p* < 0.001). Median levels of vigorous-intensity PA and total PA were significantly higher for boys than for girls (*p* < 0.05 for both outcomes); whereas levels of ST were similar (273.7 versus 220.0 min/day for boys and girls, respectively; *p* > 0.05).

**Conclusions:**

These findings highlight the high prevalence of poor dietary intake and physical inactivity in adolescents from Latin American countries. Therefore, effective and sustainable strategies and programmes are needed that promote healthier diets, regular PA and reduce ST among Latin American adolescents.

**Trial registration:**

Clinical Trials NCT02226627. Retrospectively registered on August 27, 2014.

## Background

Obesity is a major threat to worldwide public health, through increasing the likelihood of cardiovascular disease, type 2 diabetes, hypertension, metabolic syndrome, cardiovascular disorders, stroke, respiratory disease and cancer [1]. Almost three quarters of all non-communicable disease (NCD) deaths (28 million), and the majority of premature deaths (82%), occur in low- and middle-income countries, inhibiting economic and social growth [2, 3]. In conjunction with rapid demographic changes, Latin American countries (LACs) are facing a fast nutritional transition [4]. Both demographic and nutritional changes have taken place at different rates across LACs. These are associated with an increase in urbanization and the adoption of westernized lifestyles. This has led to higher levels of sedentary behaviours (SB) such as excessive sitting time (ST), lack of physical activity (PA) and poor dietary patterns characterised by excessive energy intake [5, 6].

Poor diet and lack of PA are key risk factors for increasing and alarming levels of obesity in LACs [7, 8]. However, most evidence to date has only been available from adult populations. Therefore, there is a lack of evidence about these risk factors among adolescents in the region [8, 9]. Previous studies conducted in high-income countries have provided evidence about how lifestyle behaviours and high adiposity levels in early life, including adolescence, is associated with cardiometabolic and cardiovascular risk factors in middle and later life [10–12]. Increasing the surveillance on dietary, PA, and ST patterns in adolescents using standardized methods could provide key information for the design and implementation of public health policies aiming to prevent cardiovascular risk factors and NCDs in LACs [13]. The purpose of the current study, therefore, was to investigate anthropometry, dietary intake, PA, and ST patterns in adolescents aged 15–17 years from eight LACs.

## Methods

### Study design

The Latin American Study of Nutrition and Health / Estudio Latinoamericano de Nutrición y Salud (ELANS) is a cross-sectional, multi-national survey conducted in eight LACs (Argentina, Brazil, Chile, Colombia, Costa Rica, Ecuador, Peru and Venezuela). The study was conducted over a period of 6 months (September 2014 to February 2015). The rationale and study design are reported in more detail in a previous publication [14]. The ELANS protocol was approved by the Western Institutional Review Board (#20140605) and is registered at ClinicalTrials.gov (#NCT02226627). The ethical review boards of each participating institution also approved each site-specific protocol. All study countries adhered to standardized study protocols for interviewer training, fieldwork, data collection and management, including quality control procedures.

### Participants

**Fig. 1:**
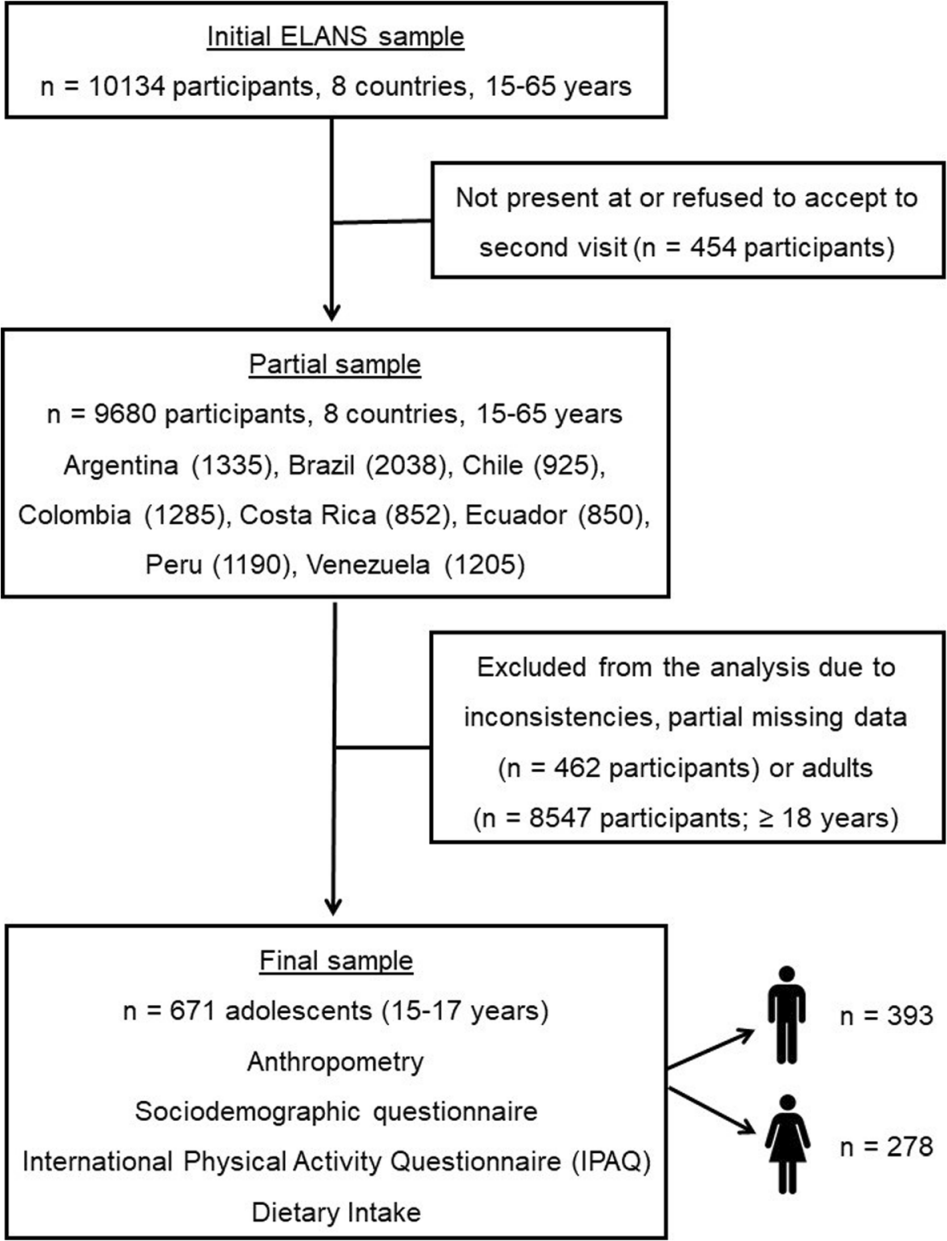
Flow diagram of the study participants in the Latin American Study of Nutrition and Health (ELANS)

Out of 10,134 individuals aged 15–65 years initially sampled, 671 were adolescents aged 15–17 years (41.4% girls) and were eligible for inclusion in the current study (Fig. 1). Exclusion criteria included pregnant and lactating girls, individuals with major physical or mental impairments, adolescents without consent from a parent or legal guardian, individuals living in residential settings other than a household, and individuals who were unable to read.

### Anthropometric data

Body weight (kg) was measured with a calibrated electronic scale (Seca®, Hamburg, Germany) with an accuracy of 0.1 kg. Body height (cm) was measured with a portable stadiometer with an accuracy of 0.1 cm. Measurements were taken during inspiration, with the base of the stadiometer lightly touching the upper reaches of the head and with the participant’s head on the Frankfort Plane [15]. Body mass index (BMI; kg/m^2^) was derived from height and weight. BMI standard deviation (SD) scores were derived using the age- and sex-specific World Health Organization (WHO) growth reference for school-aged children, which were classified into four categories of nutritional / BMI status as follows: underweight (< −2SD), eutrophic (−2SD ≥ to ≤1SD), overweight (1SD > to ≤2SD), and obese (> 2SD) [16].

In accordance with WHO recommendations [17], waist circumference (WC) was measured with an inelastic tape to the nearest 0.1 cm and was categorized as above or below thresholds (central obesity) based on reference data by sex, age and ethnicity for adolescents compiled by Katzmarzyk and colleagues [18]. Each measurement was repeated to ensure accuracy, and the average was used for statistical analyses. If the two readings differed by more than the previously established set point, then a third measurement was taken.

### Dietary intake

Dietary intake was assessed using two 24-h food recalls (24-HR) applied on non-consecutive days, with a minimum of three and a maximum of eight non-consecutive days. The 24-HR food recall method has inherent strengths including: 1) the instrument collects actual food intake on specific days, 2) recall memory is less, compared to other methods such as a Food Frequency Questionnaire; and 3) usual or habitual intake can be estimated if the instrument is repeated on the same participants [14]. For these reasons, the 24-HR food recall method has been widely used in population-based studies, such as the US National Health and Nutrition Examination Survey (NHANES), the Korean National Health and Nutrition Examination Survey (KNHANES), and the European Food Consumption Validation (EFCOVAL) study [19–21].

The protocol using 24-HR recall was administered by trained interviewers face-to-face using the Multiple Pass Method [22–24]. The households were supervised by trained nutritionists who were also responsible for converting the measures into grams (g) and millilitres (mL). This data was transformed into energy taking into account macro- and micro-nutrient quantities using the Nutrition Data System for Research software (NDS-R Version 2013, Minnesota University, Minnesota, USA). The quality control system and complete procedure for standardization of the food composition database are available in other published studies [14, 25].

Researchers in each LAC analysed the consistency of the food recall data by reviewing the quantities of total energy intake expressed as kilocalories (kcal) and as macronutrients including protein, carbohydrate, added sugar, and fat (total and saturated) expressed as a proportion (%) of total energy intake (hereafter referred to as % TE).

As a single 24-HR recall is limited and generally inadequate for assessing the usual dietary intake of individuals, two 24-HR recalls were chosen to estimate habitual food consumption and evaluate intra-individual variability in nutrient and energy intake [26]. To assess habitual dietary intake, the Multiple Source Method (MSM) was applied. This method was chosen because of its capability for improving estimates of usual dietary intake by considering within-person variance in intake [24]. As the MSM requires at least 2 days of short-term dietary measurements, all participants in the present study provided two 24-HR food recalls on non-consecutive days. Briefly, the MSM method is a mixed model, which is comprised of three parts. Firstly, the probability of energy consumption or nutrients per day is estimated using logistic regression with participant-specific random effects (probability model). Secondly, data that has been transformed for normality (using a Box-Cox transformation) is used to estimate the usual amount of food intake on consumption days using linear regression (quantity model) with participant-specific random effects. Thirdly, the estimated usual food/nutrient intake for each participant is calculated by multiplying the probability of consumption of a food/nutrient (part 1) with the usual amount of food intake (part 2) [24]. The usual intake of protein, carbohydrate, total fat and saturated fat are estimated in grams (g); energy intake is estimated in kilocalories (kcal). The relative proportion of macronutrients and saturated fat in relation to total energy intake was calculated (% TE). Acceptable macronutrient distribution ranges (AMDR) were used to evaluate the % TE from protein, carbohydrate, total fat and saturated fat [27]. The AMDRs for macronutrients were as follows: protein (10 to 35%), total fat (20 to 35%) and carbohydrates (45 to 65%). The AMDR for saturated fat was chosen in accordance with guidelines from the Food and Agriculture Organization of the United Nations (FAO) and the WHO, which recommends maximum intake of up to 10% TE in saturated fat and added sugar [28, 29].

### Measurement of self-reported physical activity and sitting time by the International Physical Activity Questionnaire (IPAQ)

PA and ST were assessed at the second visit using a Spanish language long-form, last 7-day, self-administered version of the International Physical Activity Questionnaire (IPAQ) [30]. IPAQ is designed to assess the levels of habitual PA for individuals aged 15–69 years [31, 32]. The IPAQ contains questions on the amount of walking undertaken, and on the amount of participation in moderate (MPA) and vigorous (VPA) intensity activities during active transport and leisure-time [30].

Data were analysed in accordance with the IPAQ scoring protocol [33]. The IPAQ assesses walking separately: in line with the protocol, walking was assigned an intensity of 3.3 metabolic equivalents (METs), and all other MPA and VPA were assigned an intensity of 4.0 and 8.0 METs, respectively. Total PA (expressed as minutes per week multiplied by MET values) was derived as minutes of walking × 3.3 METs + minutes of MPA (excluding walking) × 4.0 METs + minutes of VPA × 8.0 METs. Adolescents were categorized as “meeting” (≥60 min/day) or “not meeting” (<60 min/day) moderate to vigorous intensity PA (MVPA) guidelines [34]. In addition, the IPAQ contains two items that capture ST. Participants were asked to estimate the amount of time (min/day) spent sitting at work, at home, and during leisure-time for a weekday and a weekend day, separately [35]. We added weekday and weekend day ST to calculate average daily ST (weekday ST*5 + weekend day ST*2)/7.

### Statistical analyses

Descriptive statistics (with 95% confidence intervals) were calculated using means, medians, and percentages as summary measures. Overall (i.e. across all eight LACs) and country-specific levels of anthropometry (body weight, body height, waist circumference, and BMI), dietary intake (% TE from protein, carbohydrate, added sugar, total fat and saturated fat), PA and ST were estimated by sex. Similar analyses were conducted for BMI status (% obese), excess intake of saturated fat and added sugar (>10% TE), and physical inactivity (not meeting MVPA guidelines). Normality of the continuous variables was verified using the Kolmogorov-Smirnov test. Sex differences were assessed using t-tests and Mann-Whitney tests for independent samples. Since the minutes per week spent on PA was not normally distributed, we present values for the 25th, 50th (median) and 75th percentiles. The Kruskal-Wallis test was used to compare levels of PA across the four nutritional / BMI status categories. Differences in other variables for each BMI status category were compared using the Chi-square test.

All tests of statistical significance were based on two-sided probability (*p* < 0.05). Data analyses were performed with SPSS V22 software (SPSS Inc., IBM Corp., Armonk, New York, NY, USA) [36]. Analyses were weighted, with weights calculated according to the socio-demographic characteristics, sex and region of each country [14].

## Results

Overall, the mean values of body weight, body height and BMI were 60.6 kg, 164.8 cm and 22.3 kg/m^2^ respectively. Costa Rica and Chile had the highest BMI averages (23.3 kg/m^2^ for both). Just over one-fourth of adolescents (25.4%) were overweight (17.8%) or obese (7.6%). The highest prevalence of overweightness was in Chile (25%) and the highest prevalence of obesity was in Costa Rica (17.1%). Overall, mean WC was 75.3 cm, and the highest prevalence of central obesity was observed in Costa Rica (15.7%) followed by Brazil (6.2%) (Table 1). In every country except Colombia, the prevalence of overweight or obese adolescents showed no sex difference (*p* > 0.05) (Additional file 1: Table S1).

**Table 1:** Descriptive analysis (percentage or mean and 95% confidence interval) anthropometric and dietary intake of adolescents for each Latin America country

Variables	Argentina (*n* = 89)	Brazil (*n* = 128)	Chile (*n* = 68)	Colombia (*n* = 76)	Costa Rica (*n* = 70)	Ecuador (*n* = 63)	Peru (*n* = 95)	Venezuela (*n* = 82)	Overall (*n* = 671)
Age (years) ^b^	16.0 (15.8–16.1)	15.9 (15.8–16.1)	15.8 (15.6–16.0)	16.0 (15.8–16.2)	16.2 (16.0–16.4)	15.9 (15.7–16.1)	16.0 (15.8–16.1)	15.8 (15.6–15.9)	15.9 (15.8–16.0)
Body weight (kg) ^b^	60.8 (58.7–62.8)	63.2 (60.6–65.9)	64.6 (61.8–67.9)	56.5 (54.5–58.5)	62.8 (59.3–66.1)	57.8 (54.9–60.7)	57.63 (55.6–59.7)	60.8 (57.7–64.4)	60.6 (59.6–61.7)
Body height (cm) ^b^	166.9 (165.1–168.9)	168.7 (167.1–170.2)	166.5 (164.3–168.7)	164.3 (162.6–165.9)	164.1 (162.0–166.0)	161.4 (159.4–163.6)	159.9 (158.5–161.4)	164.4 (162.5–166.2)	164.8 (164.1–165.5)
BMI (kg/m^2^) ^b^	21.8 (21.1–22.6)	22.1 (21.4–23.1)	23.3 (22.5–24.2)	20.9 (20.3–21.5)	23.3 (22.1–24.5)	22.1 (21.2–23.1)	22.5 (21.8–23.2)	22.3 (21.4–23.2)	22.3 (21.9–22.6)
BMI categories ^a^									
Underweight	10.1 (4.5–16.9)	17.2 (10.9–24.2)	4.4 (0.2–10.3)	21.1 (11.8–31.6)	17.1 (8.6–25.7)	6.3 (1.6–12.7)	6.5 (2.2–12.0)	13.4 (6.1–20.7)	12.4 (10.0–15.0)
Eutrophic	73.0 (64.0–82.0)	53.1 (44.5–61.7)	63.2 (51.5–73.5)	67.1 (56.6–77.6)	45.7 (34.3–58.6)	71.4 (60.3–82.5)	69.6 (59.8–78.3)	57.3 (47.6–68.3)	62.1 (58.4–66.2)
Overweight	10.1 (4.5–16.9)	21.1 (14.1–28.1)	25.0 (14.7–35.3)	11.8 (5.3–19.7)	20.0 (11.4–28.6)	15.9 (7.9–25.4)	18.5 (10.9–26.1)	19.5 (12.2–29.2)	17.8 (58.4–66.2)
Obese	6.7 (2.2–12.4)	8.6 (3.9–13.3)	7.4 (1.5–14.7)	0 (0)	17.1 (8.6–25.7)	6.3 (1.6–12.7)	5.4 (1.1–10.9)	9.8 (3.7–17.1)	7.6 (5.7–9.6)
WC (cm) ^b^	74.7 (72.9–76.6)	74.4 (72.3–76.6)	78.6 (76.1–81.2)	70.7 (69.1–72.7)	80.4 (77.3–83.6)	74.7 (72.7–76.9)	75.6 (73.9–77.3)	75.0 (72.5–77.7)	75.3 (74.5–76.2)
WC categories ^a^									
Below threshold	96.6 (92.1–99.8)	93.8 (89.8–97.7)	94.1 (88.2–98.5)	98.7 (96.1–100.0)	84.3 (75.7–92.9)	95.2 (88.9–99.8)	97.8 (94.6–100.0)	96.3 (91.5–100.0)	94.8 (93.1–96.4)
Above threshold	3.4 (0.1–7.9)	6.2 (2.3–10.9)	5.9 (1.5–11.8)	1.3 (0.1–5.2)	15.7 (8.6–24.3)	4.8 (0.1–11.1)	2.2 (0.1–5.4)	3.7 (0.1–7.3)	5.2 (3.7–7.0)
Energy intake (kcal/day) ^b^	2415.4 (2296.3–2534.5)	2058.2 (1934.3–2181.1)	1835.9 (1716.3–1955.4)	2351.9 (2211.8–2492.2)	1919.9 (1797.1–2042.6)	2194.6 (2075.7–2313.5)	2136.0 (2041.6–2230.5)	2093.0 (1959.2–2226.9)	2129.9 (2084.6–2175.3)
Protein (% TE) ^b^	15.4 (14.9–15.9)	16.4 (15.8–16.9)	14.6 (14.1–15.2)	14.5 (13.9–15.0)	13.7 (13.2–14.2)	15.1 (14.5–15.7)	13.9 (13.5–14.3)	15.4 (14.9–15.8)	15.0 (14.8–15.2)
Carbohydrate (% TE) ^b^	52.7 (51.4–54.1)	52.3 (51.4–53.3)	55.2 (53.9–56.5)	53.7 (52.6–54.8)	57.3 (55.9–58.7)	54.6 (53.5–55.6)	63.8 (62.8–64.8)	52.5 (51.0–53.9)	55.2 (54.7–55.7)
Added sugar (% TE) ^b^	17.9 (16.7–19.1)	14.8 (13.8–15.8)	13.1 (12.1–14.1)	11.6 (10.7–12.5)	15.9 (14.6–17.3)	10.7 (9.7–11.6)	14.3 (13.5–15.2)	14.7 (13.7–15.6)	14.3 (13.9–14.7)
Total fat (% TE) ^b^	31.9 (30.8–32.9)	31.3 (30.4–32.1)	30.1 (28.9–31.3)	31.8 (30.8–32.7)	28.9 (27.7–30.1)	30.2 (29.4–31.0)	22.2 (21.4–23.1)	32.1 (30.7–33.4)	29.8 (29.4–30.2)
Saturated fat (% TE) ^b^	11.5 (11.0–12.0)	10.0 (9.6–10.4)	11.2 (10.7–11.7)	10.8 (10.4–11.2)	8.9 (8.5–9.4)	9.1 (8.7–9.4)	6.4 (6.15–6.73)	10.1 (9.7–10.6)	9.7 (9.5–9.9)
Saturated fat (>10% TE) ^a^	69.7 (60.7–78.7)	51.6 (43.0–60.2)	69.1 (58.8–80.9)	64.5 (53.9–75.0)	31.4 (20.0–41.4)	36.5 (23.8–47.6)	1.1 (0.0–3.2)	53.7 (43.9–64.6)	46.8 (42.9–50.8)
Added sugar (>10% TE) ^a^	95.5 (91.0–98.9)	76.6 (69.5–83.6)	76.5 (66.2–85.3)	64.5 (53.9–75.0)	85.7 (75.8–92.9)	47.6 (34.9–58.7)	87.4 (80.0–93.7)	84.1 (76.8–91.5)	78.4 (75.1–81.7)

^a^percentage and 95% confidence interval;

^b^mean and 95% confidence interval;

*BMI* Body mass index, *WC* Waist circumference, *TE* Total energy

Overall, mean energy intake was 2129.9 kcal/day: the average being highest in Argentina (2415.4 kcal/day) followed by Ecuador (2194.6 kcal/day). The mean contribution of macronutrients (as a % of TE intake) was 15% TE for protein, and 55.2% TE for carbohydrates. Brazil had the highest intake of protein (16.4% TE) followed by Argentina and Venezuela (15.4% TE in both). Peru had the highest intake of carbohydrates (63.8% TE) followed by Ecuador (57.3% TE). In relation to added sugar, total and saturated fat, the mean energy contribution was 14.3% TE, 29.8% TE and 9.7% TE respectively. Overall, 46.8 and 78.4% of adolescents consumed >10% TE of calories from saturated fat and added sugar, respectively (Table 1). These proportions showed no sex differences (*p* > 0.05). The prevalence of saturated fat (>10% TE) was highest for boys in Chile (75%) and for girls in Argentina (80%). For added sugar, the prevalence (>10% TE) was highest in boys (93.2%) and girls (100%) from Argentina (Additional file 1: Table S1). Overall, the prevalence of >10% TE of calories from saturated fat was higher in girls than in boys for all four BMI categories (Additional file 1: Table S2).

In relation to time spent walking, the overall median was 15.0 min/day. Time spent walking was highest in Costa Rica (25.3 min/day). The highest levels of MPA, excluding walking, were in Chile and Ecuador (21.4 min/day for both). The highest median level of VPA was observed in Brazil (30.0 min/day). For total PA, the highest median was in Chile (1687.5 MET-min/week) followed by Ecuador (1659.0 MET-min/week). Overall, the prevalence of physical inactivity (< 60 min/day in MVPA) was 29.2%: this ranged from 15.0% in Ecuador to 41.6% in Venezuela. The median daily ST was 245.0 min/day; and the highest level was observed in Chile (330.0 min/day) (Table 2).

**Table 2:** Descriptive analysis (percentage and 95% confidence interval or median and 25th and 75 percentile) of physical activity and sitting time of adolescents for each Latin America country

Variables	Argentina	Brazil	Chile	Colombia	Costa Rica	Ecuador	Peru	Venezuela	Overall
Walking (min/day) ^b^	14.3 (8.6–30.0)	15.0 (8.6–30.0)	15.0 (10.0–30.0)	17.5 (8.9–30.0)	25.3 (8.6–35.3)	15.0 (8.6–30.0)	15.0 (8.6–28.6)	12.4 (7.5–25.0)	15.0 (8.6–30.0)
Moderate PA (min/day) ^b^	15.0 (8.5–34.3)	17.1 (7.1–30.0)	21.4 (12.8–33.2)	8.6 (3.2–21.1)	12.8 (6.4–25.5)	21.4 (10.0–43.9)	17.1 (4.3–21.8)	12.8 (6.4–25.7)	17.1 (6.6–30.0)
Vigorous PA (min/day) ^b^	25.7 (12.1–48.6)	30.0 (11.4–51.4)	21.4 (8.6–68.6)	25.6 (8.6–42.9)	25.7 (11.8–62.1)	20.3 (12.8–43.4)	17.1 (6.4–50.0)	18.6 (12.8–38.6)	25.7 (8.6–51.4)
Total PA (MET-min/week) ^b^	1137.0 (504.0–2655.0)	1125.0 (412.5–2659.0)	1687.5 (626.0–3284.2)	1053.0 (473.0–2388.5)	1343.0 (594.0–2808.0)	1659.0 (901.8–3496.5)	897.0 (396.0–2147.7)	1074.0 (321.7–2161.8)	1188.0 (480.0–2660.7)
Physical inactivity (%) ^a^	28.7 (19.5–37.9)	33.6 (25.6–41.6)	24.6 (13.8–35.4)	32.9 (23.3–43.8)	21.4 (12.9–31.4)	15.0 (6.7–25.0)	28.9 (20.0–38.9)	41.6 (31.2–53.2)	29.2 (25.8–32.9)
ST total (min/day) ^b^	240.0 (135.0–360.0)	240.0 (163.1–330.0)	330.0 (210.0–405.0)	300.0 (180.0–450.0)	240.0 (130.0–360.0)	210.0 (150.0–330.0)	270.0 (180.0–360.0)	210.0 (102.5–277.5)	245.0 (150.0–360.0)
ST (min/day) on weekdays ^b^	240.0 (120.0–360.0)	240.0 (150.0–360.0)	360.0 (172.5–480.0)	315.0 (180.0–480.0)	240.0 (120.0–420.0)	240.0 (120.0–420.0)	330.0 (240.0–480.0)	210.0 (107.5–330.0)	300.0 (140.0–420.0)
ST (min/day) on weekend ^b^	240.0 (120.0–360.0)	195.0 (120.0–360.0)	300.0 (180.0–360.0)	300.0 (142.5–420.0)	240.0 (120.0–420.0)	180.0 (120.0–240.0)	240.0 (135.0–330.0)	180.0 (105.0–255.0)	240.0 (120.0–360.0)

^a^percentage and 95% confidence interval;

^b^median and 25th and 75th percentile;

*PA* Physical activity, *MET* Metabolic equivalent, *ST* Sitting time

Differences between sexes for anthropometry, dietary intake, PA and ST by LACs are shown in Tables 3 and 4. Overall, average levels of body weight, body height and energy intake were higher in boys than in girls (*p* < 0.05). In contrast, mean BMI was higher in girls than in boys (*p* < 0.05). Overall, average levels of WC showed no sex difference. This was also the case for the mean contribution of macronutrients (as a % of TE intake) for protein, carbohydrates, added sugar, total and saturated fats (Table 3).

**Table 3:** Descriptive analysis (mean and 95% confidence interval) anthropometric and dietary intake of adolescents by sex for each Latin America country

Variables	Argentina	Brazil	Chile	Colombia	Costa Rica	Ecuador	Peru	Venezuela	Overall
Body weight (kg)									
*p*-value^1^	0.006	<0.001	<0.001	0.759	0.014	0.378	0.005	0.002	<0.001
Boys	62.9 (60.3–65.6)	66.7 (63.1–70.5)	69.1 (65.3–73.4)	56.3 (53.8–58.9)	66.8 (61.6–72.2)	59.1 (55.4–63.3)	60.4 (57.9–63.3)	65.8 (61.2–71.7)	63.6 (62.4–65.1)
Girls	56.5 (53.2–60.2)	56.6 (53.9–59.8)	58.3 (55.4–61.5)	56.9 (53.9–59.9)	58.1 (54.7–62.1)	56.5 (51.9–60.7)	54.8 (52.3–57.4)	55.1 (51.8–58.8)	56.5 (55.4–57.7)
Body height (cm)									
*p*-value^1^	<0.001	<0.001	<0.001	0.002	<0.001	<0.001	<0.001	<0.001	<0.001
Boys	171.8 (170.1–173.4)	172.7 (171.2–174.2)	172.6 (170.5–174.6)	166.3 (164.1–168.3)	170.2 (168.4–172.2)	166.0 (163.5–168.5)	165.2 (163.4–167.1)	169.3 (167.1–171.4)	169.7 (169.0–170.4)
Girls	157.2 (155.2–159.4)	161.2 (159.3–162.7)	157.8 (155.7–159.8)	161.2 (159.1–163.2)	157.1 (155.2–159.1)	156.1 (153.7–158.2)	154.7 (153.2–156.2)	158.7 (156.8–160.9)	158.0 (157.3–158.8)
BMI (kg/m^2^)									
*p*-value^1^	0.041	0.603	0.665	0.005	0.594	0.055	0.265	0.320	0.049
Boys	21.3 (20.5–22.1)	22.3 (21.2–23.5)	23.1 (22.1–24.3)	20.2 (19.6–20.9)	22.9 (21.4–24.7)	21.3 (20.4–22.3)	22.1 (21.2–22.9)	22.8 (21.5–24.4)	22.0 (21.6–22.4)
Girls	22.9 (21.6–24.3)	21.8 (20.7–23.0)	23.5 (22.2–24.9)	21.9 (20.9–22.8)	23.6 (22.1–25.2)	23.1 (21.5–24.7)	22.9 (21.9–23.9)	21.8 (20.6–23.0)	22.6 (22.2–23.1)
WC (cm)									
*p*-value^1^	0.398	0.061	0.276	0.026	0.901	0.937	0.214	0.042	0.055
Boys	75.3 (73.3–77.4)	75.9 (73.2–78.7)	29.7 (76.6–83.3)	69.2 (67.6–71.0)	80.6 (76.4–85.3)	74.6 (72.1–77.6)	76.5 (74.5–78.8)	77.6 (73.7–82.5)	76.0 (74.9–77.2)
Girls	73.6 (70.0–77.5)	71.5 (68.6–74.8)	76.9 (73.8–80.4)	73.1 (70.3–76.4)	80.2 (76.3–85.3)	74.8 (71.1–78.6)	74.5 (72.2–76.8)	72.0 (69.4–74.7)	74.4 (73.3–75.7)
Energy intake (kcal/day)									
*p*-value^1^	< 0.001	<0.001	<0.001	0.028	0.003	<0.001	0.010	0.054	<0.001
Boys	2606.8 (2483.3–2734.8)	2222.9 (2058.6–2373.7)	1991.8 (1855.3–2133.4)	2476.3 (2322.1–2644.9)	2088.3 (1952.2–2216.2)	2375.9 (2210.2–2546.4)	2250.6 (2141.8–2366.7)	2213.0 (2040.1–2388.0)	2289.7 (2231.8–2350.8)
Girls	2039.0 (1857.1–2238.4)	1754.3 (1606.2–1927.5)	1613.1 (1458.4–1778.5)	2161.3 (1938.5–2403.0)	1731.1 (1558.3–1918.7)	1982.1 (1853.3–2103.4)	2008.8 (1859.4–2153.4)	1954.0 (1799.7–2151.3)	1904.2 (1840.1–1963.8)
Protein (% TE)									
*p*-value^1^	0.988	0.512	0.271	0.508	0.467	0.687	0.051	0.926	0.458
Boys	15.4 (14.8–15.9)	16.5 (15.7–17.3)	14.4 (13.8–15.0)	14.4 (13.8–14.9)	13.5 (12.8–14.2)	15.1 (14.2–15.9)	14.2 (13.7–14.8)	15.4 (14.8–16.0)	15.0 (14.8–15.3)
Girls	15.4 (14.5–16.3)	16.1 (15.2–17.1)	15.0 (14.0–15.9)	14.7 (13.7–15.8)	13.9 (13.2–14.7)	15.3 (14.4–16.2)	13.5 (13.0–14.0)	15.3 (14.7–16.0)	14.9 (14.6–15.2)
Carbohydrate (% TE)									
*p*-value^1^	0.255	0.627	0.481	0.139	0.041	0.396	0.392	0.696	0.409
Boys	53.3 (51.9–54.8)	52.5 (51.5–53.5)	54.8 (53.2–56.5)	54.4 (53.2–55.6)	58.7 (56.5–60.7)	55.0 (53.6–56.5)	64.2 (62.7–65.7)	52.8 (51.1–54.7)	55.4 (54.8–56.1)
Girls	51.7 (49.2–54.2)	52.0 (50.3–53.8)	55.8 (53.5–57.9)	52.7 (50.7–54.8)	55.9 (54.4–57.2)	54.1 (52.6–55.6)	63.4 (62.1–64.7)	52.2 (49.9–54.5)	54.9 (54.1–55.8)
Added sugar (% TE)									
*p*-value^1^	0.952	0.319	0.960	0.705	0.065	0.483	0.156	0.084	0.352
Boys	17.9 (16.5–19.3)	15.2 (14.0–16.4)	13.1 (11.8–14.4)	11.4 (10.4–12.4)	14.7 (12.9–16.9)	10.3 (9.3–11.7)	13.7 (12.6–15.0)	13.9 (12.6–15.2)	14.1 (13.6–14.7)
Girls	17.8 (15.6–20.3)	14.1 (12.1–15.8)	13.1 (11.4–14.9)	11.8 (10.2–13.3)	17.2 (15.6–18.9)	11.0 (9.6–12.6)	14.9 (13.9–16.2)	15.6 (14.2–17.0)	14.5 (13.9–15.2)
Total fat (% TE)									
*p*-value^1^	0.152	0.304	0.186	0.173	0.049	0.388	0.064	0.643	0.191
Boys	31.3 (30.1–32.5)	30.9 (29.9–31.9)	30.8 (29.3–32.1)	31.2 (30.2–32.3)	27.8 (26.0–29.8)	29.9 (28.9–30.9)	21.5 (20.4–22.8)	31.8 (30.1–33.4)	29.5 (28.9–30.1)
Girls	32.9 (30.9–34.8)	31.8 (30.4–33.2)	29.2 (27.4–31.1)	32.6 (30.7–34.4)	30.2 (28.9–31.6)	30.6 (29.4–31.7)	23.1 (22.0–24.2)	32.4 (30.4–34.4)	30.1 (29.4–30.8)
Saturated fat (% TE)									
*p*-value^1^	0.030	0.827	0.347	0.023	0.005	0.258	0.042	0.273	0.106
Boys	11.1 (10.6–11.7)	10.0 (9.5–10.6)	11.4 (10.8–12.0)	10.4 (9.9–10.9)	8.4 (7.8–9.0)	8.9 (8.4–9.4)	6.1 (5.8–6.5)	9.9 (9.3–10.4)	9.6 (9.3–9.8)
Girls	12.3 (11.4–13.3)	10.1 (9.5–10.6)	10.9 (10.1–11.8)	11.4 (10.6–12.2)	9.7 (9.0–10.3)	9.3 (8.8–9.8)	6.7 (6.4–7.1)	10.4 (9.7–11.1)	9.9 (9.6–10.2)

^1^t-tests for independent samples; significant diference were accepted at *p* < 0.05

*BMI* Body mass index, *WC* Waist circumference, *TE* Total energy

**Table 4:** Descriptive analysis (median and 25th and 75 percentile) of physical activity and sitting time of adolescents by sex for each Latin America country

Variables	Argentina	Brazil	Chile	Colombia	Costa Rica	Ecuador	Peru	Venezuela	Overall
Walking (min/day)									
*p*-value^1^	0.292	0.672	0.759	0.996	0.805	0.017	0.277	0.154	0.544
Boys	42.8 (14.6–90.0)	20.0 (11.8–35.0)	30.0 (14.3–48.7)	15.0 (7.8–21.4)	35.0 (17.1–55.0)	20.0 (10.7–60.0)	30.0 (22.3–42.5)	10.6 (6.0–15.7)	20.0 (11.4–40.0)
Girls	17.1 (11.1–27.5)	30.0 (11.4–45.0)	8.6 (6.1–17.1)	20.3 (5.9–50.3)	25.7 (5.7–51.0)	10.3 (8.2–15.2)	10.7 (3.9–87.5)	9.6 (8.2–11.0)	12.8 (6.9–32.1)
Moderate PA (min/day)									
*p*-value^1^	0.982	0.427	0.992	0.043	0.637	0.465	0.762	0.628	0.221
Boys	25.7 (8.6–57.8)	28.6 (10.3–35.0)	19.3 (10.9–36.4)	14.3 (8.6–32.1)	11.4 (8.6–22.8)	21.4 (15.0–42.8)	10.3 (4.3–22.8)	8.6 (6.1–15.0)	17.1 (8.6–34.3)
Girls	21.4 (10.7–25.7)	17.1 (3.2–23.6)	17.1 (11.8–25.7)	3.8 (1.9–6.3)	8.6 (4.3–25.3)	15.0 (7.8–27.3)	10.7 (3.9–30.0)	7.8 (5.2–9.6)	13.9 (4.3–25.4)
Vigorous PA (min/day)									
*p*-value^1^	0.896	0.352	0.573	0.157	0.689	0.102	0.405	0.204	0.009
Boys	34.3 (18.6–51.4)	85.7 (20.7–128.6)	25.7 (11.8–120.0)	25.7 (11.4–55.7)	30.0 (3.7–57.8)	17.1 (11.1–77.1)	21.4 (8.1–53.6)	25.7 (12.8–45.0)	28.6 (11.4–60.0)
Girls	34.2 (10.7–51.1)	8.6 (5.3–69.6)	15.0 (3.2–102.8)	11.8 (2.7–30.0)	20.0 (8.6–68-0)	15.7 (7.8–20.9)	8.6 (4.3–39.6)	12.3 (9.8–15.7)	14.6 (8.0–27.8)
Total PA (MET-min/week)									
*p*-value^1^	0.251	0.131	0.137	0.515	0.029	0.034	0.002	0.019	<0.001
Boys	3450.0 (2360.0–5739.0)	5773.0 (2526.0–9485.0)	2920.5 (2110.9–8003.4)	3262.0 (1572.5–6274.3)	3099.5 (1319.2–4386.2)	2964.0 (1419.0–5982.0)	2475.2 (1394.2–5513.5)	1619.0 (1313.5–3243.0)	2997.0 (1706.5–5982.0)
Girls	2911.5 (1659.7–3735.7)	1755.0 (1164.0–5038.5)	1722.0 (825.0–6599.0)	1306.5 (1102.5–2105.6)	1513.0 (1314.0–5694.1)	1514.5 (865.6–2518.9)	2467.5 (694.2–4443.7)	1240.0 (1080.7–1405.7)	1683.0 (1068.7–3870.4)
ST total (min/day)									
*p*-value^1^	0.495	0.914	0.543	0.096	0.564	0.510	0.845	0.997	0.317
Boys	120.0 (90.0–360.0)	330.0 (202.5–392.5)	255.0 (198.7–393.7)	210.0 (75.0–510.0)	195.0 (149.6–476.2)	180.0 (120.0–255.0)	220.0 (128.1–258.7)	210.0 (62.5–262.5)	220.0 (120.0–342.5)
Girls	270.0 (142.5–352.5)	240.0 (195.0–360.0)	270.0 (172.5–420.0)	300.0 (283.1–468.7)	360.0 (90.0–510.0)	165.0 (40.0–331.9)	300.0 (238.1–390.0)	285.0 (230.2–320.7)	273.7 (172.5–367.5)
ST (min/day) on weekdays									
*p*-value^1^	0.705	0.829	0.490	0.033	0.416	0.351	0.322	0.316	0.467
Boys	180.0 (90.0–360.0)	300.0 (180.0–420.0)	360.0 (165.0–427.5)	240.0 (30.0–420.0)	180.0 (89.2–427.5)	180.0 (150.0–360.0)	285.0 (90.0–385.0)	210.0 (50.0–305.0)	240.0 (120.0–420.0)
Girls	240.0 (150.0–465.0)	240.0 (180.0–390.0)	360.0 (210.0–420.0)	450.0 (315.0–630.0)	300.0 (60.0–420.0)	210.0 (52.5–420.0)	300.0 (240.0–360.0)	270.5 (220.5–320.7)	285.0 (180.0–420.0)
ST (min/day) on weekend days									
*p*-value^1^	0.582	0.848	0.378	0.120	0.424	0.212	0.355	0.827	0.446
Boys	120.0 (60.0–180.0)	300.0 (105.0–450.0)	210.0 (135.0–315.0)	120.0 (45.0–540.0)	270.0 (150.0–525.0)	120.0 (60.0–180.0)	135.0 (67.5–221.2)	130.0 (70.0–235.0)	150.0 (90.0–270.0)
Girls	180.0 (135.0–360)	300.0 (120.0–390.0)	180.0 (135.0–420.0)	270.0 (71.2–367.5)	300.0 (120.0–600.0)	150.0 (50.0–270.0)	315.0 (213.7–420.0)	190.0 (130.5–250.4)	232.5 (120.0–360.0)

^1^Mann-Whitney test; significant diference were accepted at *p* < 0.05

*PA* Physical activity, *MET* Metabolic equivalent, *ST* Sitting time

Median levels of walking and moderate-intensity PA showed no sex differences (*p* > 0.05 for both). In contrast, median levels of vigorous-intensity and total PA were significantly higher for boys than for girls (*p* < 0.05). Levels of ST were similar between the sexes (*p* > 0.05) (Table 4). The prevalence of physical inactivity (% < 60 min/day in MVPA) was significantly higher for girls than for boys (43.7 and 19%, respectively). Physical inactivity prevalence was highest for boys in Brazil and Venezuela (26.8% in both) and was highest for girls in Venezuela (58.3%) (Additional file 1: Table S3).

In additional analyses, we examined median levels of PA and ST in the four BMI categories. Overall, and within each LAC, boys and girls had similar (p > 0.05) levels of physical inactivity and similar median levels of total PA in each BMI category (Additional file 1: Tables S3-S4). Patterns by sex and by country were less clear for the median levels of ST for each BMI category (Additional file 1: Table S4).

## Discussion

The aim of this study was to investigate anthropometry, dietary intake, PA, and ST patterns in adolescents (aged 15–17 years) from eight LACs. Overall, average levels of body weight and body height were higher in boys, whilst mean BMI was higher in girls (*p* < 0.05). Boys had a higher (*p* < 0.05) total energy intake than girls. Prevalence of TE saturated fat and added sugar (>10% TE) was higher in girls than boys (49.6% versus 44.8 and 81.7% versus 76.1%, respectively), but these differences were not statistically significant (*p* = 0.214 and *p* = 0.084 respectively). Median levels of vigorous-intensity PA and of total PA were significantly higher for boys than for girls, whilst median levels of ST were similar between both groups (220.0 and 273.7 min/day, respectively).

Acceptable macronutrient distribution ranges (AMDR) were used to evaluate the distribution of adolescents relative to the total energy intake percentage (% TE) from protein, carbohydrates and total fat [37]. Diethelm et al. [38] indicated that the percentage of the total caloric intake of macronutrients was approximately 49% TE for carbohydrates, 34% TE for total fat, and 14% TE for saturated fat among adolescents aged 15–19 years (no assessment of % TE was made for protein). López-Sobaler et al. [39] evaluated the balanced caloric intake among Spanish adolescent participants (aged 14–17 years) in the National Dietary Survey on the Child and Adolescent Population project (ENALIA). In the ENALIA study, estimated TE % was 18% (protein), 46% (carbohydrates), 34% (total fat), and the estimate for saturated fat ranged from 11.4% in girls to 12% in boys. Therefore, the relative total caloric intake of macronutrients from protein, total and saturated fat are lower among Latin American adolescents compared to their European counterparts. In the American population, estimated protein intake (% TE) of adolescent and young adult (14–18 years old) participants in NHANES was similar to the eight LACs included in our study for both sexes (boys: 16.0% NHANES versus 15.0% LACs; girls: 14.4% NHANES versus 15.0% LACs) [40].

Few studies have been conducted amongst the Latin American population in order for us to compare our findings. Results from the Brazilian Study of Cardiovascular Risks in Adolescents (ERICA) were similar to those found in the present study. The estimates in ERICA for the mean contribution of macronutrients (as a % of TE intake) from protein (15.4 and 16.3%), carbohydrate (54.0 and 53.7%), total fat (30.9 and 30.2%) and saturated fat (11.3 and 10.8%) among females and males aged 14–17 years, respectively, were close to our results [41].

The high saturated fat intake confirmed by our study should be highlighted, as we can see on a global scale that American, Latin American and European adolescent populations have a high percentage of total energy intake that comes from this nutrient [38, 39, 41]. According to the FAO and the WHO, saturated fat should provide a maximum of 10% of total energy intake; but, in the present study, higher energy intake from this nutrient was reported by 46.8% of adolescents. Both organizations emphasize that it is important to assess not only total consumed lipids but also the local availability of their fractions (i.e. % TE), in order to elaborate and provide effective dietary guidance to promote adolescent health. Another important finding is that only 21.6% of ELANS adolescents met the recommendation of consuming less than 10% of energy from added sugar [29]. Excessive sugar consumption increases the risk for obesity and several other NCDs in both adolescents and adults.

In our study, boys engaged in more PA than girls in all countries. This finding is consistent with previous studies of sex disparities in PA [42, 43]; however, the magnitude of the difference differs by PA intensity. Results from the current study show that the sex difference is greater for vigorous- than for moderate-intensity PA. Efforts should therefore be made to develop agendas that specifically target and engage girls in increasing the intensity of their physical activity; however, to date, sex differences in PA intensity has seldom been investigated in LACs.

The consistent finding, confirmed also by our study, that boys are more active than girls provides the primary rationale for many interventions targeting physical inactivity among adolescent girls. Previous literature supports the argument for sex-targeted PA interventions, because adolescent boys and girls prefer different activities, participate in PA for different reasons, and may face different barriers to engaging in PA [44]. Furthermore, issues such as the involvement of the family [45] and the perception of an unfavourable family situation, together with social roles, could explain to some extent, the lower levels of activity among adolescent girls [46]. PA interventions may also need to target girls at an earlier chronological age than boys, considering that, on average, girls mature 2 years earlier than boys [42].

We found in our study that median levels of ST were similar for both sexes. This finding is also consistent with other studies [6, 47]. Furthermore, the most notable finding of the current study was that median levels of ST did not vary by BMI status for either sex. This finding differs from our hypothesis and does not agree with the few studies that compared SB levels according to BMI status in young people [48, 49]. For example, Compernolle et al. [48] found differences in SB between overweight/obese and normal weight adolescents. The high amount of time spent daily on SB may be concerning, as previous literature [50] suggests that high levels of PA may not protect adolescents from the risks to health due to excessive amounts of time spent on SB.

A number of studies have compared self-reported data on PA with device-based methods such as accelerometry [30, 51, 52] and PA related energy-expenditure through the doubly-labelled water method [53]. The majority of studies showed positive but moderate associations between reported and device-based methods [30, 51]. Previous studies estimated correlations of 0.23–0.40 between the self-reported data and accelerometer-assessed MVPA [51, 54, 55]. Questionnaires remain the most feasible method to assess levels of PA at a population level due in part to expensive costs and high respondent burden associated with using device-based methods within large-scale health examination surveys [56].

The present study has several strengths. Our study fills a gap in the evidence because to date no studies have presented a multi-country assessment of dietary intake and PA patterns among Latin American adolescents. A further strength is its comprehensive assessment of dietary intake through using two non-consecutive days of 24-h dietary recall. Also, the estimates of usual energy and macronutrient intake were based on statistical methods performed to appropriately adjust for intra-individual variability; and such procedures allowed the removal of extreme values [57]. Our study is the first to evaluate PA and ST patterns in Latin American adolescents using a standardized methodology across a consortium of several participating countries. This study thus provides a unique Latin American dataset that will enable wider cross-country comparisons and therefore expand the existing literature.

Some limitations of the present study are also recognized. ELANS employed a cross-sectional design, precluding inferences about causality. In addition, since the ELANS data represent the dietary intake of urban adolescents in eight LACs, care must be taken to extrapolate these findings to other countries in South and Central America. Although data from the rural population was not collected in this study, it should be highlighted that the majority of the populations studied currently live in urban regions (64 to 92%) [58]. Misreporting could have altered the estimated levels of dietary intake presented. Under-reporting of healthy diets occurs in most adult populations, especially in women and in those with higher BMI. As reported in the literature [59], under-reporting could be attributable to participant’s denial, a low ability to accurately report dietary intake, or due to social desirability bias. Despite these limitations, these data are the best currently available to evaluate dietary energy intake among Latin American adolescents. Finally, the current study did not include adolescents older than 17 years because the MVPA guidelines (≥60 min/day) are for 5–17 year olds. The limited evidence regarding the validity of the IPAQ instrument among adolescents means that caution must be exercised when interpreting our findings on self-reported PA and ST.

## Conclusions

In conclusion, standardized data from the ELANS showed that the average levels of daily mean energy intake was higher in boys than in girls, but that the distribution of total energy intake across different macronutrients was similar between the sexes across the eight participating LACs. Overall, prevalence of physical inactivity was significantly higher in girls than boys.

Further research is needed to explore possible reasons for the sex differences in PA detailed in our analyses. Future studies with larger samples of children and adolescents are needed to obtain a more representative understanding of the energy intake, PA and SB of adolescents in the Latin American region.

## Supplementary information

**Additional file 1: Table S1.** Prevalence of nutritional status (%) and of total energy (>10%) from saturated fat and added sugar of adolescents by sex for each Latin America country. **Table S2.** Prevalence of total energy (>10%) from saturated fat and added sugar of adolescents by sex and by nutritional status for each Latin America country. **Table S3.** Prevalence (%) of physical inactivity by sex and by nutritional (BMI) status for each Latin America country. **Table S4.** Descriptive analysis (median and 25th and 75 percentile) of total physical activity (MET-min/week) and sitting time of adolescents by sex and by nutritional status for each Latin America country.

## Data Availability

The dataset used and analysed during the current study are available from the corresponding author on reasonable request.

## Abbreviations

AMDR: Acceptable macronutrient distribution ranges
BMI: Body mass index
ELANS: Latin American Study of Nutrition and Health
ENALIA: National Dietary Survey on the Child and Adolescent Population
ERIKA: Brazilian Study of Cardiovascular Risks in Adolescents
FAO: Food and Agriculture Organization of the United Nations
IPAQ: International Physical Activity Questionnaire
LACs: Latin American countries
Min: Minutes
MPA: Moderate-intensity physical activity
MSM: Multiple source method
MVPA: Moderate to vigorous intensity physical activity
NCDs: Non-communicable diseases
NHANES: National Health and Nutrition Examination Survey
PA: Physical activity
SB: Sedentary behaviours
ST: Sitting time
TE: Total energy
USDA: United States Department of Agriculture
VPA: Vigorous-intensity physical activity
WC: Waist circumference
WHO: World Health Organization
