# Supplementary material for: Anthropometry, dietary intake, physical activity and sitting time patterns in adolescents aged 15–17 years: an international comparison in eight Latin American countries
Source: BMC Pediatr. 2020 Jan 21;20:24. doi: 10.1186/s12887-020-1920-x (PMC6971876; doi:10.1186/s12887-020-1920-x)
Supplement: Supplementary file 1 — Additional file 1: Table S1. Prevalence of nutritional status (%) and of total energy (>10%) from saturated fat and added sugar of adolescents by sex for each Latin America country. Table S2. Prevalence of total energy (>10%) from saturated fat and added sugar of adolescents by sex and by nutritional status for each Latin America country. Table S3. Prevalence (%) of physical inactivity by sex and by nutritional (BMI) status for each Latin America country. Table S4. Descriptive analysis (median and 25th and 75 percentile) of total physical activity (MET-min/week) and sitting time of adolescents by sex and by nutritional status for each Latin America country. [file 12887_2020_1920_MOESM1_ESM.docx]

**Table S1.** Prevalence of nutritional status (%) and of total energy (>10%) from saturated fat and added sugar of adolescents by sex for each Latin America country.

| Variables | Argentina | Brazil | Chile | Colombia | Costa Rica | Ecuador | Peru | Venezuela | Overall |
| --- | --- | --- | --- | --- | --- | --- | --- | --- | --- |
| **BMI categories** |  |  |  |  |  |  |  |  |  |
| p-value^1^ | 0.473 | 0.214 | 0.946 | **0.019** | 0.317 | 0.420 | 0.964 | 0.556 | 0.059 |
| Boys |  |  |  |  |  |  |  |  |  |
| Underweight | 11.9 | 18.1 | 5.0 | 30.4 | 24.3 | 5.9 | 6.4 | 9.1 | 14.3 |
| Eutrophic | 74.5 | 51.8 | 65.0 | 63.0 | 40.5 | 79.4 | 70.2 | 56.8 | 62.1 |
| Overweight | 6.8 | 18.1 | 22.5 | 6.6 | 16.3 | 11.8 | 17.0 | 22.7 | 15.1 |
| Obese | 6.8 | 12.0 | 7.5 | 0 | 18.9 | 2.9 | 6.4 | 11.4 | 8.5 |
| Girls |  |  |  |  |  |  |  |  |  |
| Underweight | 6.7 | 15.6 | 3.6 | 6.7 | 9.1 | 6.9 | 6.7 | 18.4 | 9.7 |
| Eutrophic | 70.0 | 55.6 | 60.7 | 73.3 | 51.5 | 62.1 | 68.9 | 57.9 | 62.2 |
| Overweight | 16.7 | 26.6 | 28.6 | 20.0 | 24.2 | 20.7 | 20.0 | 15.8 | 21.6 |
| Obese | 6.6 | 2.2 | 7.1 | 0 | 15.2 | 10.3 | 4.4 | 7.9 | 6.5 |
| **WC categories** |  |  |  |  |  |  |  |  |  |
| p-value^1^ | 0.219 | 0.534 | 0.712 | 0.213 | 0.903 | 0.462 | 0.144 | 0.645 | 0.391 |
| Boys |  |  |  |  |  |  |  |  |  |
| Below threshold | 98.3 | 92.8 | 95.0 | 100.0 | 83.8 | 97.1 | 100.0 | 95.5 | 95.4 |
| Above threshold | 1.7 | 7.2 | 5.0 | 0 | 16.2 | 2.9 | 0 | 4.5 | 4.6 |
| Girls |  |  |  |  |  |  |  |  |  |
| Below threshold | 93.3 | 95.6 | 92.9 | 96.7 | 84.8 | 93.1 | 95.6 | 97.4 | 93.9 |
| Above threshold | 6.7 | 4.4 | 7.1 | 3.3 | 15.2 | 6.9 | 4.4 | 2.6 | 6.1 |
| **Saturated fat (>10% TE)** |  |  |  |  |  |  |  |  |  |
| p-value^1^ | 0.130 | 0.768 | 0.210 | 0.073 | 0.061 | 0.205 | 0.340 | 0.246 | 0.214 |
| Boys | 64.4 | 50.6 | 75.0 | 56.5 | 21.6 | 29.4 | 2.0 | 47.7 | 44.8 |
| Girls | 80.0 | 53.3 | 60.7 | 76.7 | 42.4 | 44.8 | 0 | 60.5 | 49.6 |
| **Added sugar (>10% TE)** |  |  |  |  |  |  |  |  |  |
| p-value^1^ | 0.144 | 0.284 | 0.811 | 0.867 | **0.011** | 0.268 | 0.097 | 0.067 | 0.084 |
| Boys | 93.2 | 79.5 | 77.5 | 65.2 | 75.7 | 41.2 | 82.0 | 77.3 | 76.1 |
| Girls | 100.0 | 71.1 | 75.0 | 63.3 | 97.0 | 55.2 | 93.3 | 92.1 | 81.7 |
| ^1^ : Chi-Square test;  BMI: body mass index; WC: waist circumference; TE: total energy. | | | | | | | | | |

**Table S2.** Prevalence of total energy (>10%) from saturated fat and added sugar of adolescents by sex and by nutritional status for each Latin America country.

| Variables | Argentina | Brazil | Chile | Colombia | Costa Rica | Ecuador | Peru | Venezuela | Overall |
| --- | --- | --- | --- | --- | --- | --- | --- | --- | --- |
| **Saturated fat (>10% TE)** |  |  |  |  |  |  |  |  |  |
| Boys |  |  |  |  |  |  |  |  |  |
| p-value^1^ | 0.522 | 0.255 | 0.834 | 0.811 | 0.777 | 0.442 | 0.173 | 0.294 | 0.241 |
| Underweight | 42.9 | 40.0 | 100.0 | 50.0 | 11.1 | 50.0 | 0 | 25.0 | 37.5 |
| Eutrophic | 68.2 | 53.5 | 73.1 | 58.6 | 26.7 | 33.3 | 0 | 60.0 | 48.3 |
| Overweight | 75.0 | 66.7 | 77.8 | 66.7 | 16.7 | 0 | 12.5 | 30.0 | 45.8 |
| Obese | 50.0 | 30.0 | 66.7 | --------- | 28.6 | 0 | 0 | 40.0 | 33.3 |
| Girls |  |  |  |  |  |  |  |  |  |
| p-value^1^ | 0.360 | 0.218 | 0.266 | 0.622 | 0.465 | 0.400 | --------- | 0.099 | 0.657 |
| Underweight | 100.0 | 71.4 | 0 | 100.0 | 0 | 50.0 | --------- | 71.4 | 55.6 |
| Eutrophic | 71.4 | 40.0 | 52.9 | 77.3 | 47.1 | 55.6 | --------- | 59.1 | 47.4 |
| Overweight | 100.0 | 66.7 | 75.0 | 66.7 | 50.0 | 16.7 | --------- | 83.3 | 55.0 |
| Obese | 100.0 | 100.0 | 100.0 | --------- | 40.0 | 33.3 | --------- | 0 | 44.4 |
| **Added sugar (>10% TE)** |  |  |  |  |  |  |  |  |  |
| Boys |  |  |  |  |  |  |  |  |  |
| p-value^1^ | 0.691 | 0.472 | 0.151 | 0.995 | **0.014** | 0.398 | 0.252 | **0.034** | 0.380 |
| Underweight | 100.0 | 80.0 | 100.0 | 64.3 | 55.6 | 0 | 100.0 | 25.0 | 69.6 |
| Eutrophic | 90.9 | 76.7 | 84.6 | 65.5 | 93.3 | 40.7 | 75.8 | 84.0 | 76.4 |
| Overweight | 100.0 | 93.3 | 66.7 | 66.7 | 100.0 | 50.0 | 100.0 | 70.0 | 83.1 |
| Obese | 100.0 | 70.0 | 33.3 | --------- | 42.9 | 100.0 | 100.0 | 100.0 | 72.7 |
| Girls |  |  |  |  |  |  |  |  |  |
| p-value^1^ | --------- | 0.912 | 0.640 | 0.445 | 0.123 | 0.192 | 0.693 | 0.281 | 0.463 |
| Underweight | 100.0 | 71.4 | 100.0 | 100.0 | 100.0 | 50.0 | 100.0 | 100.0 | 88.9 |
| Eutrophic | 100.0 | 72.0 | 76.5 | 63.6 | 100.0 | 66.7 | 90.3 | 90.9 | 82.7 |
| Overweight | 100.0 | 66.7 | 62.5 | 50.0 | 100.0 | 50.0 | 100.0 | 100.0 | 78.3 |
| Obese | 100.0 | 100.0 | 100.0 | --------- | 80.0 | 0 | 100.0 | 66.7 | 72.2 |
| ^1^ : Chi-Square test;  BMI: body mass index; TE: total energy. | | | | | | | | | |

**Table S3.** Prevalence (%) of physical inactivity by sex and by nutritional (BMI) status for each Latin America country.

| Variables | Argentina | Brazil | Chile | Colombia | Costa Rica | Ecuador | Peru | Venezuela | Overall |  |
| --- | --- | --- | --- | --- | --- | --- | --- | --- | --- | --- |
| **Physical inactivity (%) by sex** | | |  |  |  |  |  |  |  |  |
| p-value^1^ | 0.065 | **0.027** | **0.007** | **0.005** | **0.004** | **0.004** | **0.020** | **0.005** | **<0.001** |  |
| Boys | 22.4 | 26.8 | 12.8 | 20.5 | 8.1 | 3.0 | 17.8 | 26.8 | 19.0 |  |
| Girls | 41.4 | 46.5 | 42.3 | 51.7 | 36.4 | 29.6 | 40.0 | 58.3 | 43.7 |  |
| **Physical inactivity (%) by sex and by nutritional (BMI) status** | | | | | | |  |  |  |  |
| Boys |  |  |  |  |  |  |  |  |  |  |
| p-value^1^ | 0.176 | 0.417 | 0.691 | 0.656 | 0.084 | 0.058 | 0.100 | 0.286 | 0.850 |  |
| Underweight | 42.9 | 40.0 | 0 | 23.1 | 0 | 0 | 0 | 0 | 21.8 |  |
| Eutrophic | 18.6 | 27.9 | 12.0 | 21.4 | 0 | 0 | 13.3 | 34.8 | 17.6 |  |
| Overweight | 50.0 | 20.0 | 11.1 | 0 | 16.7 | 25.0 | 50.0 | 11.1 | 21.4 |  |
| Obese | 0 | 11.1 | 33.3 | --------- | 28.6 | 0 | 0 | 40.0 | 18.8 |  |
| Girls |  |  |  |  |  |  |  |  |  |  |
| p-value^1^ | 0.387 | 0.415 | 0.198 | 0.148 | 0.125 | 0.768 | 0.972 | 0.265 | 0.224 |  |
| Underweight | 0 | 28.6 | 100.0 | 50.0 | 0 | 50.0 | 33.3 | 33.3 | 30.8 |  |
| Eutrophic | 50.0 | 56.0 | 33.3 | 61.9 | 52.9 | 23.5 | 38.7 | 61.9 | 47.9 |  |
| Overweight | 20.0 | 40.0 | 62.5 | 16.7 | 12.5 | 33.3 | 44.4 | 50.0 | 36.2 |  |
| Obese | 50.0 | 0 | 0 | --------- | 40.0 | 50.0 | 50.0 | 100.0 | 47.1 |  |
| ^1^ : Chi-Square test; | | | | | | | | | | |

**Table S4.** Descriptive analysis (median and 25^th^ and 75 percentile) of total physical activity (MET-min/week) and sitting time of adolescents by sex and by nutritional status for each Latin America country.

| Variables | Argentina | Brazil | Chile | Colombia | Costa Rica | Ecuador | Peru | Venezuela | Overall |
| --- | --- | --- | --- | --- | --- | --- | --- | --- | --- |
| **Total PA (MET-min/week)** | |  |  |  |  |  |  |  |  |
| Boys |  |  |  |  |  |  |  |  |  |
| p-value^1^ | 0.517 | 0.483 | 0.682 | 0.809 | 0.914 | 0.264 | 0.276 | 0.680 | 0.739 |
| Underweight | 2290.0 (816.7-2472.0) | 960.0 (426.5-3045.0) | 1323.9 (264.0-2108.0) | 1450.0 (537.0-247.0) | 1902.0 (906.9-2708.2) | 4493.5 (3588.0-5.980.0) | 975.0 (462.0-1380.0) | 1398.0 (960.0-2980.4) | 1455.0 (594.0-2662.5) |
| Eutrophic | 1638.0 (504.0-2940.0) | 1470.0 (462.0-2419.5) | 2198.2 (1181.2-4287.0) | 1920.0 (539.0-5321.0) | 2226.0 (958.5-4104.5) | 2982.0 (1419.0-4468.5) | 1533.0 (867.0-4409.5) | 1814.2 (742.5-2953.5) | 1911.0 (790.5-3525.7) |
| Overweight | 792.0 (324.0-3236.0) | 2160.0 (526.2-2997.0) | 1191.0 (506.5-3326.2) | 1690.0 (330.0-2890.2) | 3852.0 (805.5-5074.0) | 1687.5 (730.9-6561.4) | 468.0 (247.5-1907.9) | 1429.0 (709.5-2564.7) | 1507.7 (521.8-3276.4) |
| Obese | 993.5 (609.6-2871.7) | 1438.5 (834.0-7008.0) | 3045.0 (660.0-4130.0) | --------- | 1912.0 (198.0-4948.5) | --------- | 3114.4 (899.0-4908.4) | 1257.0 (371.2-5342.2) | 1832.0 (577.9-3689.2) |
| Girls |  |  |  |  |  |  |  |  |  |
| p-value^1^ | 0.234 | 0.296 | 0.228 | 0.343 | 0.136 | 0.984 | 0.947 | 0.380 | 0.515 |
| Underweight | 885.0 (810.0-921.0) | 662.0 (420.7-5065.5) | --------- | --------- | 1971.0 (758.0-2809.3) | 798.0 (462.0-2108.0) | 1440.0 (132.0-2890.5) | 495.0 (192.0-1290.5) | 810.0 (478.5-2025.0) |
| Eutrophic | 1215.0 (297.0-2318.2) | 688.5 (181.9-2362.5) | 1626.0 (479.2-2948.5) | 516.0 (317.0-1553.2) | 693.0 (247.5-1513.0) | 1320.0 (570.0-1879.5) | 537.0 (247.5-1173.7) | 569.2 (188.6-1239.0) | 697.5 (279.7-1825.5) |
| Overweight | 693.0 (330.0-3276.0) | 1233.0 (321.7-3965.0) | 746.2 (214.5-1358.0) | 611.2 (259.5-1918.9) | 813.0 (606.4-4716.1) | 1090.0 (715.5-1826.2) | 590.3 (412.5-762.7) | 297.0 (93.0-1528.0) | 720.0 (396.0-1680.0) |
| Obese | 594.0 (495.0-680.2) | --------- | --------- | --------- | 396.0 (132.0-1063.5) | 1341.9 (198.0-2150.8) | 1191.0 (396.0-2380.0) | --------- | 544.5 (346.5-2019.0) |
| **ST total (min/day)** | |  |  |  |  |  |  |  |  |
| Boys |  |  |  |  |  |  |  |  |  |
| p-value^1^ | 0.457 | 0.244 | 0.458 | 0.784 | 0.512 | 0.396 | 0.644 | 0.999 | 0.609 |
| Underweight | 450.0 (237.5-495.0) | 285.0 (195.0-450.0) | 157.5 (60.0-226.0) | 210.0 (172.5-375.0) | 270.0 (157.5-352.5) | 127.5 (60.0-237.5) | 330.0 (240.0-410.0) | 187.5 (165.0-220.0) | 255.0 (180.0-365.0) |
| Eutrophic | 240.0 (112.5-345.0) | 240.0 (180.0-330.0) | 315.0 (217.5-360.0) | 285.0 (86.2-510.0) | 240.0 (142.5-300.0) | 292.5 (150.0-360.0) | 255.0 (180.0-336.2) | 172.5 (74.4-255.0) | 240.0 (150.0-348.7) |
| Overweight | 202.0 (71.2-299.7) | 210.0 (45.0-270.0) | 360.0 (90.0-405.0) | 270.0 (190.0-389.0) | 210.0 (179.5-397.5) | 292.5 (243.7-375.0) | 330.0 (170.6-533.7) | 195.0 (37.5-281.2) | 240.0 (149.8-337.5) |
| Obese | 240.0 (183.7-375.0) | 322.5 (63.7-645.0) | 270.0 (120.0-380.0) | --------- | 210.0 (105.0-420.0) | --------- | 300.0 (180.0-420.0) | 205.0 (110.0-292.5) | 270.0 (120.0-381.7) |
| Girls |  |  |  |  |  |  |  |  |  |
| p-value^1^ | 0.123 | 0.397 | 0.551 | 0.391 | 0.247 | 0.739 | 0.114 | 0.261 | 0.680 |
| Underweight | 270.0 (90.0-320.0) | 270.0 (157.5-386.2) | --------- | --------- | 285.0 (20.0-410.0) | 157.5 (45.0-410.0) | 300.0 (285.0-480.0) | 140.0 (130.0-230.0) | 285.0 (135.0-370.0) |
| Eutrophic | 285.0 (195.0-390.0) | 210.0 (131.2-412.5) | 345.0 (210.0-450.0) | 332.0 (240.0-397.5) | 240.0 (120.0-450.0) | 180.0 (150.0-300.0) | 255.0 (165.0-360.0) | 569.2 (188.5-1239.0) | 243.7 (163.1-367.5) |
| Overweight | 90.0 (60.0-180.0) | 305.0 (217.5-435.0) | 315.0 (225.0-408.7) | 480.0 (367.5-513.7) | 1175. (63.0-292.5) | 285.0 (33.7-361.2) | 225.0 (135.0-347.6) | 297.0 (93.0-1528.5) | 240.0 (90.0-363.5) |
| Obese | 210.0 (90.0-290.0) | --------- | --------- | --------- | 300.0 (255.0-405.0) | 240.0 (210.0-340.0) | 428.0 (360.0-530.0) | --------- | 300.0 (210.0-337.5) |
| ^1^ : Kruskal-Wallis test;  PA: physical activity; MET: metabolic equivalent; ST: sitting time. | | | | | | | | | |
